# Transmitted blood infections and emerging vector-borne diseases in blood donors in northern Portugal

**DOI:** 10.1016/j.htct.2025.103734

**Published:** 2025-02-08

**Authors:** Ana Mota, Margarida Fonseca Cardoso

**Affiliations:** a Serviço de Imuno-Hemoterapia da Unidade Local de Saúde Santo António, Porto, Portugal; b Escola Superior de Saúde, Universidade Fernando Pessoa, Porto, Portugal; c Environment & Healthy Life Styles da TL7 do RISE Health, Universidade Fernando Pessoa, Porto, Portugal; d ICBAS - Instituto de Ciências Biomédica Abel Salazar, Universidade do Porto, Porto, Portugal; e CIIMAR, Universidade do Porto, Porto, Portugal

**Keywords:** Blood transfusion, Blood donors, Mandatory tests, Infectious diseases, Emerging infectious diseases, Vectors

## Abstract

**Background:**

Screening of transfusion-transmissible infectious agents of blood components is carried out in order to guarantee the safety of the transfusion process. The objective of this investigation was to characterize cases positive for transfusion-transmissible infectious agents in blood donations in the North of Portugal.

**Method:**

Data from 2010 to 2022 of the Local Health Unit-Santo Antonio were used for this study. In specific epidemiological situations, malaria, Chagas disease and West Nile virus were screened.

**Main results:**

Over 12 years, the health unit, received 137,751 donations with 108 positive tests. The proportions of human immunodeficiency viruses, syphilis, human hepatitis viruses type B and C varied between 0 and 44/100,000 donations. In this period, two cases of malaria were detected in 2020–2021, and 21 were detected in 2022 corresponding to 52.1/1000 donations screened. In 2022, two cases of Chagas disease and no cases of West Nile virus were detected.

**Conclusion:**

These results highlight the importance of a rigorous investigation at the time of donation in which the donor's history, including origin and movement in areas of greater geographic risk, are assessed. The recent and increasing detection of cases of malaria and Chagas disease confirms the presence of emerging infectious diseases transmitted by vectors, including mosquitoes, in blood donors. The increased risk of vector-borne diseases in Europe is a public health problem and represents a new challenge in screening donations.

## Introduction

Blood is a vital element with a therapeutic use that, until today, cannot be substituted. In this sense, blood donation is a solidarity and voluntary act that definitely contributes to saving lives. As blood is a biological product and, therefore, not without risks, it is necessary to use all available means to reduce the possible transmission of infection.^1^

Screening for transfusion-transmissible infectious (TTI) agents of blood components is essential to ensure the safety of the transfusion process. Regarding the risk of transmission of infections, until recently the main concern was infection with human immunodeficiency viruses type 1 or 2 (HIV 1/2) and human hepatitis viruses type B (HBV) and C (HCV).^2^ Climate change and globalization, with the possibility of rapid access to more distant parts of the world, appears to be changing the initial profile of blood-borne viral diseases.^1^^,^^3^^,^^4^ The European tendency for high temperature peaks as well as periods of drought with severe and increasingly long and hot summers, creates ideal conditions for the invasion of mosquitoes normally associated with tropical temperatures.^3^ It is known that many tropical diseases associated with transmission by vectors, such as mosquitoes, are endemic in some parts of Africa, the Americas and Asia, causing severe illness in more than one billion people around the world. Several species with vectorial capacity, capable of spreading diseases such as Chikungunya, Dengue fever, Yellow fever, Zika, West Nile virus, malaria and Chagas disease, have been observed in Europe.^5^^,^^6^

The HIV epidemic had a major impact on the blood supply at the end of the 20th century, with the first case associated with transfusion dating back to 1982.^7^, ^8^, ^9^ Over the last few decades, the transmission of infection associated with the transfusion process has progressively decreased, making its risk residual.^10^ The selection of potential blood donors has become more rigorous, either by addressing medical history and risk behaviors with a more detailed and targeted questionnaire, or by introducing new serological and molecular tests.

In the 1960s, more than 30% of patients who received multiple transfusions developed post-transfusion hepatitis, 25% of which were caused by HBV. With the implementation of sensitive tests and new policies on voluntary blood donation, there was a significant reduction in the incidence of the disease,^11^^,^^12^ partly also due to the measures taken due to the HIV infection epidemic.

In Portugal, according to national legislation, in accordance with European directives,^13^ the analysis of donations of whole blood or blood components includes, and is mandatory, the search for HBsAg, anti-HBc, anti-HCV and anti-HIV 1/2 antibodies. Nucleic acid tests (NATs) for HIV, HBV and HCV must also be carried out, as well as testing for antibodies of human T-lymphotropic virus types I and II (HTLV 1/2) in all first donations, in donors from areas of high prevalence and in those whose sexual partners or parents come from these areas. Because it is related to high-risk behaviors and because it is considered a risk factor for other sexually transmitted infectious diseases, a serological study for syphilis is carried out. Additional analyzes are recommended for certain components and donors in specific epidemiological situations, such as malaria, Chagas disease and West Nile virus.^4^^,^^14^

The infectious disease caused by the severe acute respiratory syndrome coronavirus 2 (SARS-CoV-2), known as coronavirus disease 2019 (COVID-19), has brought a major challenge to medicine globally, and in particular to transfusion medicine.^1^ A decrease in the number of donations was seen, partly due to prophylactic isolation measures (lockdown) that restricted people's travel to transfusion medicine services, as well as the public's fear of contracting the infection while traveling to hospital units. Clinical blood needs were unpredictable, in part due to the cancellation of elective surgeries and non-urgent interventions, while blood demand for emergency situations and patients on chronic transfusion support remained relatively unchanged.^15^ In Portugal, since the state of emergency was declared on March 18, 2020, a contingency plan was adopted that was capable of guaranteeing the sustainability, safety and supply of blood and its components. Compared to 2019, in 2020 there was a 7% reduction in the total number of donations. However, blood reserves always remained at sufficient levels to meet the needs of patients.^16^ The Association for the Advancement of Blood and Biotherapies (AABB), the Centers for Disease Control and Prevention (CDC), and the World Health Organization did not recommend additional measures to be implemented in the screening of blood donations. During the pandemic period, in Portugal, as in the rest of Europe, all donations remained in quarantine for 14 days, in order to enable their discard in the event of a positive result for SARS-CoV-2 infection.^1^^,^ ^17^

The objective of this investigation was to characterize cases with a positive result for TTI that require mandatory screening in blood donations in a population of volunteer donors in the North of Portugal.

## Materials and methods

This is a retrospective study of blood donations carried out in the Immunohemotherapy service of the Local Health Unit (LHU)-Santo António from January 1, 2010 to December 31, 2022. Data collection was carried out using the blood management computer database. Viral markers for HIV, HBV, HCV, and syphilis were evaluated in all donors; HTLV 1/2 research was carried out only for the donor's first donation. Informed consent regarding the donation comes from completing a survey that is initially filled out by the donor and validated by the clinician during a consultation. This survey contains information about travel. In this situation, the clinician, with the support of the Portuguese blood donation center (IPST) website, assesses the geographic risk, and depending on the risk zone, chooses to make a request for tests for malaria, Chagas and West Nile virus. To study this 12-year time segment, four periods were defined: 2010–2017, 2018–2019, 2020–2021, 2022:

- 2010–2017: corresponds to the start of the definitive computerization of the donor process.
- 2018–2021 was divided into two periods due to the COVID-19 pandemic, despite there being no changes in terms of screening technology. The period 2018–2019 corresponds to the two years that preceded the pandemic and 2020–2021 covers the two years of high incidence of Sars-Cov2.
- The 4th period corresponds only to 2022, given the limited data available.

The study focused on donors with positive results for TTI, in which true positives (TP) were identified. The definitions of TP results related to the TTIs are presented below:

- HIV 1/2: repeatedly positive chemiluminescence results with positivity in additional Western-Blot or Innolia tests or positive result for NAT.
- HBV: chemiluminescence result repeatedly positive for HBsAg or positive result for NAT. Anti-HBc positivity leads to the study of anti-HBs titers. It is considered a past infection if anti-HBc is positive with HBsAg and NAT being negative. Until October 2014, the donation was accepted if the anti-HBs value was greater than 10 IU/mL. After 2014, the donation began to be accepted for a value equal to or greater than 100 IU/mL.^14^ This procedure is performed on all donations.
- HCV: repeatedly positive chemiluminescence results with positivity in supplementary tests by Recombinant ImmunoBlot Assay (RIBA) or Innolia or positive result for NAT.
- HTLV 1/2: repeatedly positive chemiluminescence results with positivity in additional tests by Innolia.
- Syphilis: an active infection was considered when it corresponds to the presence of a positive chemiluminescence test (combined treponemal test - IgM and IgG), confirmed by a positive non-treponemal test, such as Venereal Disease Research Laboratory (VDRL). Samples that are enzymatic immunoassay (EIA) positive and VDRL negative indicate a past infection with syphilis, and in this case, the donation is discarded and the donor is suspended.
- Malaria: search for antibodies against Plasmodium, associated with the onset of malaria through repeatedly positive serological results with confirmation by molecular testing. The screening is carried out by the IPST. Positive serology, even with a negative molecular test, does not allow acceptance of the donation. In Portugal, and with regard to blood donations, the malaria test is requested for donors with a history of malaria and donors born in or coming from an endemic area (according to decree-law 267/2007, later revised and amended by decree 185/2015).^13^ A positive serological test for malaria leads to the entire blood unit being discard, even if the molecular biology test for malaria is negative. According to the European guidelines the donor is temporarily suspended and the serological test is repeated after three years to assess whether the result remains positive. The donor can only be accepted if the serological test is negative. If the molecular biology test is positive, the donor is referred for a specialist consultation. The test used for malaria screening was the EIA of Bio-Rad, designed to detect antibodies. The molecular test used was the VIASURE Malaria Real-Time PCR Detection Kit (CerTest, Biotec), designed for the qualitative and quantitative detection of DNA of *Plasmodium* species, including the main malaria species that infect humans: *Plasmodium falciparum, Plasmodium vivax, Plasmodium malariae, Plasmodium ovale, Plasmodium knowlesi*.
- Chagas disease: the serological test should be requested for donors born in or coming from an endemic area. The Alinity i Chagas test is a chemiluminescent microparticle immunoassay (CMIA) used for the qualitative detection of antibodies against *Trypanosoma cruzi* (according to decree-law 267/2007, and later revised and amended by decree 185/2015).^13^ A repeatedly positive serological test leads to the entire blood unit being discarded and the elimination of the donor. If the test is positive, the donor is referred for a specialist consultation.
- West Nile Virus: was only screened when there was information about a geographical risk, mainly in southern Europe. Donations from donors coming from those areas were evaluated by a molecular test, the Cobas® WNV test, a real-time polymerase chain reaction (PCR) test to detect West Nile virus. The screening is carried out by the IPST.

All donations with a TP result for TTI were discarded. All non-negative analytical results that were not confirmed as TP were considered false positives. Donations with a false positive result, even with a negative NAT test, were also discarded.

For each donation, the age, sex and birthplace of the respective donors were recorded.

### Statistical analysis

Distribution by sex and place of birth are reported as percentages, and age is reported as a mean ± standard deviation. Positive tests and the number of confirmed TP were counted for each infection in each of the four time periods. Based on these values, the confirmation rates for each of the infections are reported by calculating the percentage of TP in positive tests. In the total number of donations, the prevalences of positive tests and TPs were also estimated, dividing the number identified by the total number of donations and multiplying by 100,000 or by 1000 depending on the total number of donations. The Statistical Package for Social Sciences (SPSS) version 26 was used for statistical analysis.

## Results

Between 2010 and 2022, the LHU-Santo António received 137,751 donations. This study is focused on 455 donations with positive results for TTIs; 54.1% corresponded to male donors, with an average age of 42.2 (± 13.1) and 1.5% from outside Portugal.

Table 1 presents the positive tests obtained in donations collected between 2010 and 2022 for HIV 1/2, syphilis, HCV, HBV, HTLV 1/2, malaria and Chagas disease, and the corresponding number of tests confirmed as positive. A total of 19 individuals were tested for West Nile virus, 11 in 2016, four in 2017, two in 2018, and two in 2019, but none tested positive.

**Table 1:** Number of positive results for transfusion-transmissible infectious agents, confirmed number of true positives (TP), and estimated prevalence in the total blood donations for each period.

	Period	Total donations	Positive results	Proportion of positive results	TP	%TP /Positive	Proportion of TP
HIV 1/2	2010–2017	93,667	49	52.3/100,000	8	16.33%	8.5/100,000
	2018–2019	18,268	2	10.9/100,000	0		
	2020–2021	17,958	5	27.8/100,000	2	40.00%	11.1/100,000
	2022	7858	2	25.5/100,000	0		
Syphilis	2010–2017	93,668	137	146.3/100,000	41	29.93%	43.8/100,000
	2018–2019	18,268	20	109.5/100,000	8	40.00%	43.8/100,000
	2020–2021	17,958	12	66.8/100,000	6	50.00%	33.4/100,000
	2022	7858	4	50.9/100,000	2	50.00%	25.5/100,000
HCV	2010–2017	93,668	110	117.4/100,000	3	2.73%	3.2/100,000
	2018–2019	18,268	14	76.6/100,000	1	7.14%	5.5/100,000
	2020–2021	17,958	14	78.0/100,000	0		
	2022	7858	6	76.4/100,000	0		
HBV	2010–2017	93,668	22	23.5/100,000	9	40.91%	9.6/100,000
	2018–2019	18,268	6	32.8/100,000	0		
	2020–2021	17,958	7	39.0/100,000	1	14.29%	5.6/100,000
	2022	7858	6	76.4/100,000	1	16.67%	12.7/100,000
HTLV 1/2^a^	2010–2017	33,167	13	39.2/100,000	1	7.69%	3.0/100,000
	2018–2019	1840					
	2020–2021	2860	1	35.0/100,000	0		
	2022	1080					
Malaria^b^	2010–2017						
	2018–2019						
	2020–2021	235	2	8.5/1000	2	100%	8.5/1000
	2022	403	21	52.1/1000	21	100%	52.1/1000
Chagas^b^	2010–2017						
	2018–2019						
	2020–2021	451	0				
	2022	292	2	6.8/1000	2	100%	6.8/1000
West Nile	2010–2017	15	0				
Virus^b^	2018–2019	4	0				
	2020–2021						
	2022						

HIV 1/2: Human immunodeficiency virus types 1 or 2; HBV: human hepatitis virus type B; HCV: human hepatitis virus type C; HTLV 1/2: human T-lymphotropic virus types I and II.

a The HTLV 1/2 test is only performed on the first donations.

b Test performed according to the assessment of the geographic risk.

It was from 2020 onwards that screening for malaria and Chagas disease became recommended for donors from risk areas or traveling to areas of the world where there are endemic levels for these two diseases. In the years 2020–2022, 638 requests for malaria screening and 743 for Chagas disease were analyzed. In the period from 2020 to 2022, 23 donors tested positive for antibodies against Plasmodium and had a confirmatory molecular test for malaria of which the majority were from Portugal, with only one from Brazil, one from India, four from Mozambique and one from Poland. This situation implies the suspension of donations for 3 years. It should be noted that there were positive and confirmed tests for Chagas disease only in 2022 (two cases), both from Portugal.

## Discussion

This investigation arose with the purpose of characterizing cases of transfusion-transmissible infections that require the mandatory screening of blood donations from volunteer blood donors in the North of Portugal. The study covered 12 years (2010–2022) of donations at the LHU-Santo António, with a change in the profile of transmissible infectious diseases identified during this period. The study shows a small number of donors who were confirmed as having HIV 1/2, HCV, HBV or HTLV 1/2, ranging between 0 and 13 per 100,000 donations, and syphilis, ranging between 25 and 44 per 100,000. These reduced values contrast with the observed proportion of positive results in donations screened for malaria, which increased by around 100 times compared to previous results, with 8.5 and 52.1 per 1000 registered in 2020–2021 and 2022, respectively, or even Chagas with 6.8 per 1000 in 2022. In this period there was a total of 19 individuals tested for West Nile virus, but all of them tested negative.

During the pandemic period (2020–2021), there was a drop in blood donations around the world, although there were no records of transfusion transmission of COVID-19.^1^ In Portugal^16^ there was a reduction of around 4% of donations compared to 2018–2019. In the LHU-Santo António this reduction was around 2%. This difference was due to a policy to raise awareness in the Immunohemotherapy Service among all the LHU-Santo António staff. Until the start of the pandemic, the institution's professionals were not motivated to donate blood. This criterion took into account the greater exposure of these professionals to viral diseases transmitted by blood. The significant decrease in the number of donations from regular donors led to a campaign to motivate all professionals to donate, appealing to and focusing on professionals who provide administrative services. This procedure has been in force since 2020, which has made more donors loyal. In terms of impact, it can be seen that when the institution's staff became part of the immunohemotherapy donors, there were no relevant changes in the detection of TTIs.

Although HIV is no longer considered an epidemic, it continues to be a subject of concern to ensure blood quality.^1^ In the last 12 years, the number of TP for HIV has been very low, just ten donations. The most recent data on the Portuguese population point to a reduction in the number of new HIV cases, however the rates recorded in Portugal are among the highest in Europe.^18^ For donors in Portugal, both prevalence and incidence decreased between 2016 and 2020, but an increase was recorded in 2021.^16^

In the case of syphilis infection, the test currently used allows the identification of both acute infections and past and resolved infections. In the case of active syphilis infections, there was a significant decrease in the number of cases at the LHU-Santo António over the years under study, however, in all periods analyzed, syphilis was identified in the donations screened. Data from the National Serological Survey from 2015 to 2016 for the Portuguese population^19^ point to a seroprevalence of 2.4%. A study for the city of Rome describes a decline in the cases of sexually transmitted diseases, especially syphilis for the spring 2020 period with the strict lockdown.^20^ Similarly, for Madrid there was a decrease in sexually transmitted diseases in the first half of 2020, when compared with the same period in 2019**.** All these results suggest that the reduction in sexually transmitted diseases during the COVID-19 pandemic reflects the decrease in sexual contacts due to social distancing.^21^^,^^22^ Despite the convergence of data, it is considered that this interpretation should be cautious, since, in this pandemic period, many people were not followed up in hospitals for diagnosis.

In the case of HCV infection, no case was confirmed in the donations tracked from 2020 onwards, with only three cases confirmed in the period 2010–2017 and one in 2018–2019. In these two periods, the estimated prevalences were 3.2 and 5.5 per 100,000, respectively. The 2018–2019 values are close to the data published for blood donors in Portugal in 2018 and 2019, 4.43 and 7.48/100,000 respectively. For the period 2010–2017, the estimated prevalence in the LHU-Santo António donors is below that of national donors, which is around 10/100,000.^16^

Regarding HBV infection, nine cases were confirmed as positive in the first period from 2010 to 2017 corresponding to 9.6 per 100,000 donations, zero in 2018–2019, one case in 2020–2021 and one in 2022. This downward trend was reported nationally,^16^ and it would be expected given the mandatory vaccination of newborns since 2000.

In the 12 years of the study, only one HTLV 1/2 infection was found in the period 2010–2017, which confirms the low endemicity of this infection in Portugal.^23^

The second millennium brought new routines to blood banks, as a result of new epidemics and emerging diseases attributed to climate change and migration.^1^^,^^4^^,^^6^ The LHU-Santo Antonio began to include screening for diseases such as West Nile virus, and more recently research for malaria and Chagas disease. In a total of 638 tests for Plasmodium carried out between 2020 and 2022, 23 positive results were confirmed. In 2020 and 2021, the number of reported cases of malaria in Portugal were 59 and 79, respectively,^24^ however these values may be below the real figure due to underreporting^25^ and the fact that cases can only be detected in situations of acute illness. Until the first half of the 20th century, malaria was one of the main causes of deaths in Portugal; it was considered eradicated in 1958. It initially predominated in urban areas and became an increasingly rural disease, with a special focus on agricultural areas of rice, where it became endemic, due to the presence of the mosquito that transmits malaria.^26^

Regarding Chagas disease, in 743 test requests there were two positive cases. This confirms the presence in Portugal of tropical diseases associated with transmission by vectors, in blood donors, as has been observed in the general European population,^5^^,^^6^^,^^24^^,^^27^ confirming the importance of a rigorous investigation at the time of the donation in which the donor's history is assessed, including travel for leisure or work to areas of greater geographic risk for these diseases.^4^^,^^28^

## Conclusion

This epidemiological study allowed the investigation of the occurrences and significant changes of diseases transmitted by blood in donations from a population of donors in the North of Portugal over 12 years (2010–2022). The study puts into perspective and confirms the need to annually monitor donations in Portugal, paying special attention to emerging infectious diseases that have already started to be part of the screening panel, initially with testing for the West Nile virus and, currently, with testing for malaria and Chagas disease. It is necessary to adopt the World Health Organization recommendations^27^ for the risk of Dengue fever, Zika and Chikungunya outbreaks on the European continent, as a result of the climate crisis that facilitates the spread of mosquitoes that carry diseases with a higher incidence in the southern hemisphere.

The increase in the average life expectancy of the population, the low birth rate, the increasingly strict criteria in the selection of donors, as well as the inherent complexity of surgical and oncological procedures are factors that, as a whole, increase the need for blood. In Portugal, in the last decade the aging index has been steadily worsening. Since 2000, the number of elderly people has surpassed the number of young people in Portugal. All of these factors contribute to the imbalance between blood consumption and blood supply. This aging also extends to donors, which is why donation motivation campaigns among younger people (universities, sports associations and similar organizations) is a serious bet to attract donors who can improve this situation. It is urgent to invest in strategies that^29^ retain existing donors and implement new ones that attract more people to donate blood.

On the other hand, the 2021 census indicates that the population of foreign nationals living in Portugal will be around 800 to 900 thousand people^30^ and although in practice it is not possible to reverse the aging pyramid, they alert us to the need to a rigorous screening of 'new and emerging' viral diseases transmitted by blood, often associated with the movement of people for work or leisure.^31^

The results obtained show the need to combine the promotion of the appeal for blood donation with the attention and expansion of the type of tests for diseases transmitted by blood, fundamental to guarantee the safety of the transfusion process, not only in Europe but in countries outside Europe. Testing updates must follow international information on new epidemiological events and migratory flows, always maintaining hospitality and clinical care as an unavoidable good practice for the entire population, including that of blood donors.

## Funding

The authors received no specific funding for this work.

## Conflicts of interest

There are no conflicts identified.
